# Diversity of Functionally Distinct Clonal Sets of Human Conventional Memory B Cells That Bind Staphylococcal Protein A

**DOI:** 10.3389/fimmu.2021.662782

**Published:** 2021-04-28

**Authors:** Emily E. Radke, Zhi Li, David N. Hernandez, Hanane El Bannoudi, Sergei L. Kosakovsky Pond, Bo Shopsin, Peter Lopez, David Fenyö, Gregg J. Silverman

**Affiliations:** ^1^ Department of Medicine, New York University Grossman School of Medicine, New York, NY, United States; ^2^ Department of Biochemistry and Molecular Pharmacology, New York University Grossman School of Medicine, New York, NY, United States; ^3^ Institute for Systems Genetics, New York University Grossman School of Medicine, New York, NY, United States; ^4^ Institute of Genomic and Evolutionary Medicine, Temple University, Philadelphia, PA, United States; ^5^ Department of Pathology, New York University Grossman School of Medicine, New York, NY, United States

**Keywords:** single-cell sorting, antigen-specific, superantigen, single-cell sequencing, *Staphylococcus aureus*, Staphylococcal Protein A, SpA, human memory B cells

## Abstract

*Staphylococcus aureus*, a common cause of serious and often fatal infections, is well-armed with secreted factors that disarm host immune defenses. Highly expressed *in vivo* during infection, Staphylococcal protein A (SpA) is reported to also contribute to nasal colonization that can be a prelude to invasive infection. Co-evolution with the host immune system has provided SpA with an Fc-antibody binding site, and a Fab-binding site responsible for non-immune superantigen interactions *via* germline-encoded surfaces expressed on many human BCRs. We wondered whether the recurrent exposures to *S. aureus* commonly experienced by adults, result in the accumulation of memory B-cell responses to other determinants on SpA. We therefore isolated SpA-specific class-switched memory B cells, and characterized their encoding VH : VL antibody genes. In SpA-reactive memory B cells, we confirmed a striking bias in usage for VH genes, which retain the surface that mediates the SpA-superantigen interaction. We postulate these interactions reflect co-evolution of the host immune system and SpA, which during infection results in immune recruitment of an extraordinarily high prevalence of B cells in the repertoire that subverts the augmentation of protective defenses. Herein, we provide the first evidence that human memory responses are supplemented by B-cell clones, and circulating-antibodies, that bind to SpA determinants independent of the non-immune Fc- and Fab-binding sites. In parallel, we demonstrate that healthy individuals, and patients recovering from *S. aureus* infection, both have circulating antibodies with these conventional binding specificities. These findings rationalize the potential utility of incorporating specially engineered SpA proteins into a protective vaccine.

## Introduction

*Staphylococcus aureus* (*S. aureus*) is a common commensal and opportunistic pathogen that is a frequent cause of community- and hospital-acquired diseases, including skin and soft tissue infections (SSTI), pneumonia, bacteremia, and endocarditis (1). Alarmingly, *S. aureus* clinical isolates are also increasingly resistant to β-lactams and other antibiotics (2–4). Yet, despite attempts with many different Staphylococcal protein and polysaccharide antigens, there is no approved preventative vaccine (5, 6). Together these factors have contributed to the mounting societal burden of *S. aureus* infections, and the urgency for enhancing our understanding host-pathogen relationship with this microbial pathogen.

*S. aureus* is a cunning invader with many aspects of pathogenicity remaining poorly understood. Indeed, *S. aureus* chronically colonizes an estimated 20% of healthy adults, while the remainder are likely colonized intermittently (7–11). Limited cutaneous infections afflict everyone at one time or another, and although invasive infections are less common, some individuals suffer recurrent invasive infections (12, 13). In general, invasive infections result in substantial morbidity and mortality, and are associated with release of a multitude of exotoxins that can act as a preemptive attack on host barriers and immune defenses (14). A predominance of evidence indicates that in most individuals, recovery from infection is not accompanied by persistent enhanced immune defenses against future *S. aureus* infection (15–19).

During invasive infection, Staphylococcal protein A (SpA) is one of the most abundantly expressed *S. aureus* virulence factors (20, 21) and it is reported to also be required for host nasal mucosal colonization (22–24). It is hypothesized that SpA coats the bacterial surface due to covalent linkage to the cell wall, and it is routinely cleaved and released locally during infection (21, 25, 26). SpA produced during infection has been shown to correlate with greatly suppressed immune responses to many Staphylococcal antigens (18, 27). SpA is believed to impact host defenses by several different mechanisms. The IgG-antibody Fcγ-binding site of SpA contributes to impaired host defenses in part by blocking effective antibody-mediated opsonophagocytic killing function (28–32).

SpA also has a separate immunoglobulin Fab-binding site, which is responsible for low-affinity non-immune oligomeric binding interactions with Fab of soluble immunoglobulins (Igs) and membrane-associated B cell antigen receptors (BCR) (21, 33, 34). These binding interactions are mediated by germline-encoded contact sites on specific VH regions (35), which are inherently different than the hypervariable loops responsible for responses selected during the somatic adaptive immune response (36, 37). Through these non-immune BCR interactions, SpA has the toxin-like properties of a B-cell superantigen (21, 22, 38, 39). Mouse infection models suggest SpA interferes with the generation of long-lived plasma cells that would otherwise augment defense from reinfection (18).

Despite the potentially high prevalence of B cells that can interact with the superantigen Fab-binding site, we reasoned that recurrent exposure to this ubiquitous opportunistic pathogen may still result in immune recognition of additional determinants on SpA. Enhancing human host responses to such postulated antigenic determinants during an infection could therefore potentially facilitate efficient clearance of this staphylococcal factor, and abrogate the capacity of SpA to adversely affect host B cell defenses. Such active immune responses could represent an Achilles heel for the toxin.

To test our hypothesis, we directly investigated the immunogenetic basis for recognition by human peripheral conventional CD27-bearing memory B cells of SpA determinants. For this purpose, we developed an integrated pipeline for selection and recovery of SpA-specific B cells, which was followed by isolation, cloning and sequence analysis of natively paired VH : VL antibody genes. These antibody genes were reconstituted as recombinant IgG for further testing of reactivity and specificity. Results from studies of recombinant IgG with memory B-cell BCR were compared to those with polyclonal circulating IgG from a range of *S. aureus-*infected and healthy donors. Our findings confirm that responses to SpA are dominated by binding to the small site linked to B-cell superantigen properties. In addition, we found that a limited but significant proportion of human memory B cells, and recirculating IgG antibodies, recognize determinants on SpA that are independent of the Fab-binding responsible for non-immune interactions. Collectively, our studies rationalize a practical strategy for redirecting and strengthening responses towards these other SpA determinants. This approach should potentially neutralize the immunomodulatory properties deriving from the superantigen and Fc-binding functions of SpA, which confound host defense and the capacity for augmenting persistent protective antibody responses against this common microbial pathogen.

## Materials and Methods

### SpA Structural Visualization

**Figure 1:**
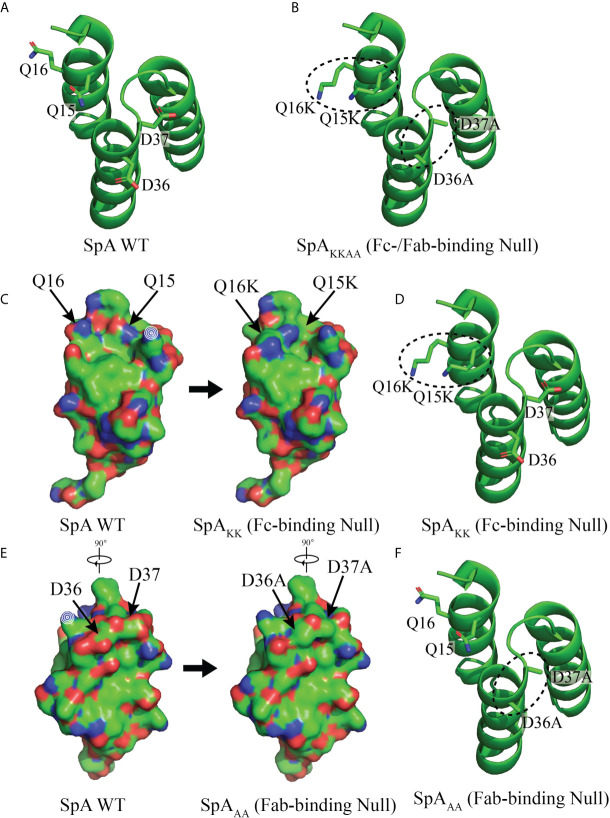
Structure of Domain D of Staphylococcal protein A (SpA) WT and mutants. **(A)** Models of the structure of WT SpA with side chains of residues Q15, Q16, D36 and D37, shown with the peptide backbone that forms a triple alpha helical bundle. **(B)** SpA_KKAA_ contains both sets of mutated residues, Q15K, Q16K, D36A and D37A to form a mutant with both Fab and Fc binding interactions abrogated. **(C)** Residues Q15 and Q16, that contribute to the Fc binding interaction (40, 46) and are mutated, Q15K and Q16K, to form the SpA_KK_ mutant with abrogated Fc binding. Little or no effect on protein folding is predicted. Mutated residues are indicated with arrows and changes in surface exposure are shown between the WT structure at left and with residue mutations Q15K and Q16K at right. **(D)** Peptide backbone with mutated Q15K and Q16K residues circled. **(E)** Residues D36 and D37 that contribute to the Fab-binding interactions (34). Mutations D36A and D37A, form the SpA_AA_ mutant with abrogated Fab-binding. Little or no effect on protein folding is predicted. Mutated residues are indicated with arrows and changes in surface exposure are shown between the WT structure at left and with residue mutations D36A and D37A at right. **(F)** Peptide backbone with mutated D36A and D37A residues circled. Modelling depiction generated with PyMOL (ref) using PDB: 1DEE.

PyMOL™ (Intel Inc) was used to visualize Domain D of WT SpA (PDB: 1DEE) for peptide backbone **Figure 1A**, amino acid side chains and the overall predicted solvent-exposed surface.

### Engineering and Expression of SpA Variants Devoid of Fc- and/or Fab- Binding Activity

For the study of BCR-mediated binding of SpA, replacement mutations were introduced at positions that mediate Fcγ-binding interactions (40) (i.e., Q15K, Q16K and an additional N34A) in a SpA D domain, termed SpA_KK_. To confirm that an antibody recognizes a “conventional” determinant on the protein, additional replacement mutations were introduced to disrupt the reported Fab-binding site (i.e., Q15K, Q16K, D36A and D37A) in a SpA D domain, termed SpA_KKAA_ as previously described (27). To emulate the natural oligomeric structure, the mutant domains were generated as a pentamer. Constructs were then synthesized and inserted into the pET-15b that introduces a His-tag at the amino terminus. The BL21 (DE3) bacteria strain was transfected then expanded under antibiotic selection and IPTG induction. Recombinant pentameric mutant SpA was recovered from lysate by passage under optimized conditions using a HisTrap HP column and a HiPrep 26/60 Sephacryl S-300 HR size exclusion column (GE Healthcare). Purity was documented by SDS-PAGE.

### Generation of SpA and Control Tetramers

Adapting a validated tetramer-based flow cytometric protocol (31, 41), tetramers were generated by incubating biotinylated pentameric mutant SpA_KK_ in a 4:1 molar ratio with streptavidin-conjugated to R-phycoerythrin (SAV-PE) (Biolegend) on ice for 2 hours. A control reagent was generated using biotinylated control protein, human serum albumin (HSA) (Sigma), similarly incubated with streptavidin conjugated to a PE-Alexa Fluor 647 dual dye (SAV-PE*AF647) (Thermo Fisher). Aggregates were removed from tetramers by centrifugation in a tabletop microcentrifuge for one hour at 4°C. Homogeneity and >95% purity were documented by SDS-PAGE and size fractionation.

### Antigen-Specific B Cell Recovery by Cell Sorting

PBMCs from a healthy adult donor were collected and B cells were enriched using a B cell Isolation Kit (Miltenyi). Cell viability of >97% was confirmed at each procedural step with a Muse Cell Analyzer (EMD Millipore Corp).

**Figure 2:**
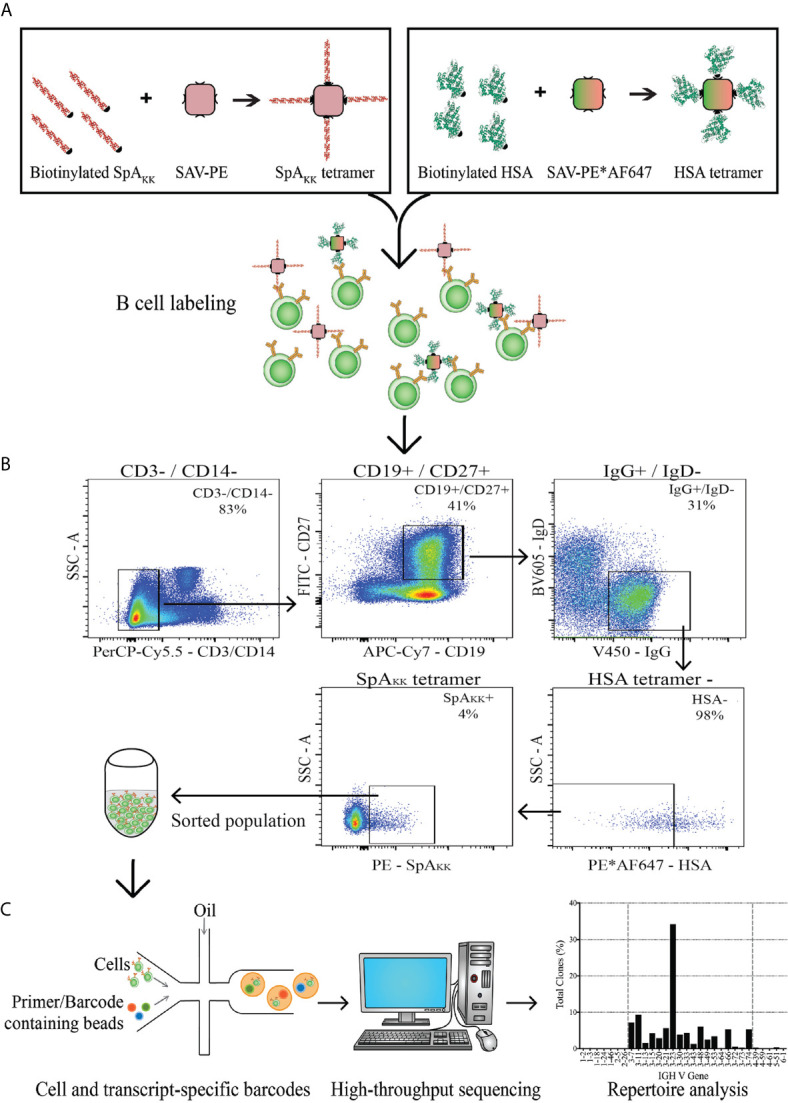
Depiction of strategy for memory B cell sorting and BCR transcript sequencing. **(A)** PBMCs from a healthy donor were labeled with either SpA_KK_ or control HSA-tetramers. **(B)** The memory B cell compartment positive for the SpA_KK_ tetramer was identified and sorted. **(C)** The sorted population was collected, with single cell analysis that incorporates unique barcodes and high-throughput sequencing. Reads of BCR transcripts were analyzed for antibody gene usage and repertoire characteristics.

For our sorting strategy, non-specific binding was blocked by including a monoclonal murine IgG1 antibody before staining with flow cytometry reagents (**Supplementary Table 1** **)**. Following the gating scheme in **Figure 2B**, we isolated SpA_KK_-binding CD19+ CD27+ IgG+ IgD- B cells that did not bind to the control tetramer, which also removed cells reactive with PE and AF647 fluorochromes, streptavidin, and non-specific binders. Cell sorting was performed using a FACSAria IIuSORP instrument (Becton Dickinson).

### Recovery of Natural VH : VL Paired Genes of Individual SpA-Binding Memory B Cells

Antibody gene sequences that encoded for membrane-associated BCR from freshly isolated individual B cells were recovered using the 10X Chromium system (10X Genomics) which, facilitated amplification of antibody genes from individual sorted B cells (**Figure 2C**, SRA: PRJNA694313). In brief, individual antigen-specific B cells were encapsulated into nanoliter-sized droplets termed Gel Bead in emulsion (GEM), which facilitated the lysis of single cells and reverse transcription of polyadenylated mRNA with introduction of barcodes in individual oligonucleotides. Sequencing of antibody gene libraries was completed in a single bulk reaction, using a standard next generation sequencing method (MiSeq instrument, Illumina). To ensure sequence fidelity, hundreds of reads were obtained for each barcode. Loupe VDJ Browser (10X Genomics), IMGT^®^/HighV-QUEST (42) and BRepertoire (43) were used to assess reading frames and assign closest germline genes.

The compiled database of complete antibody VH genes from the sort was also analyzed with a sequence dissimilarity matrix with the longest common substring distance implemented as the dissimilarity measure. This approach in part enabled quantification of unpaired amino acid residues between each pair of unique reads, without affecting the deduced primary amino sequence of these compared reads. Each deletion or insertion was treated equally with a weight of 1. The sum of all weights was defined as the distance between each of the two reads. The dissimilarity matrix derived from pairwise comparisons of all the VH sequences was then used as input for class multidimensional scaling to depict each cell in a two-dimension plot. Statistically significant differences were measured by Mann-Whitney analysis, additional analysis was performed with custom R script and Prism 7.0 (GraphPad).

### Generation and Sequencing of RACE-Based Ig Gene Libraries

Adapting a validated previously reported protocol (44), we generated unbiased γ rearrangement transcript libraries from class-switched B cells from healthy adult blood donors. RNA was first isolated from PBMCs using the PAXgene Blood RNA Kit (PreAnalytiX). cDNA of γH chain-specific transcripts were then amplified using the SMARTer^®^ RACE 5’/3’ kit (Takara) then amplified with specific oligonucleotide primers that included Illumina barcodes (44). Libraries were sequenced (MiSeq, Illumina) and data analyzed, as described above.

### Synthesis and Purification of Recombinant Antibodies

Using a validated protocol to express recombinant antibodies, selected VH : VL paired sequences were codon optimized and these genes were synthesized, then sub-cloned into the pcDNA3.4 expression vector (GenScript). Expi293F cells were transfected. After six days in culture, recombinant antibodies were purified from supernatants using RoboColumn Eshmuno A (Sigma-Aldrich). Purity of >98% was documented by SDS-PAGE gel under reducing and non-reducing conditions.

### Collection of Blood Samples From *Staphylococcus aureus*-Infected and Control Volunteers

Human subjects were enrolled and informed consent was obtained, as previously described (20, 45), following IRB approval under institutional supervision at two university medical centers: Bellevue Hospital and NYU Tisch Hospital, and serum samples were collected from patients with Skin and Soft Tissue Infection (SSTI) and uninfected adult controls.

### Direct and Competition Antibody and Sera Binding ELISA

Binding interactions were assessed by ELISA, with microtiter wells precoated overnight with either rSpA WT (Repligen), mutant SpA_KK_, mutant SpA_KKAA_, tetanus toxoid (Enzo) or SARS-CoV-2 spike (Sino Biological). Wells were then blocked with 2% BSA/PBS. In parallel assays, different recombinant IgG1 antibodies or human sera were then incubated at a range of concentrations. Wells were washed and antibody binding detected using goat anti-human IgG Fc specific-HRP (Jackson ImmunoResearch). Wells were measured using TMB substrate (BioLegend), and read with a Flexstation 3 Multi-Mode Microplate Reader (Molecular Devices).

For competition binding studies, the previously described ELISA protocol was used with SpA_KK_ pre-coated on an ELISA plate at 1 µg/mL. Each mAb at a concentration of 1 µg/mL was pre-incubated with a range of concentrations of SpA_KKAA_ from 5 µg/mL to 0.16 µg/mL, or no SpA_KKAA_, before addition to the SpA_KK_ precoated then blocked wells. Levels of Ig binding were then determined. Similarly, sera were pre-incubated at a concentration of 1:5000 with the SpA_KKAA_ before addition to SpA_KK_ wells.

## Results

### Isolation of Human Memory B Cells That Bind SpA

To accurately study the BCR repertoire expressed by human class-switched peripheral memory B cells that bind SpA, we utilized a SpA variant with ablating mutations in the Fc-binding site, which was termed SpA_KK_ (**Figures 1C, D**, Methods 2.2, and data not shown) (46). Using a fluorochrome-labeled tetramer of this variant SpA_KK_, we then designed a flow cytometric sorting panel that excluded non-specific binders, including cells that bound the fluorochrome or streptavidin **(** **Figure 2A**). From a PBMC sample from a healthy adult donor without history of recent *S. aureus* infection, using our sorting panel, we captured conventional memory CD27+ IgG+ B cells and gated for binding activity of the SpA_KK_-tetramer (**Figure 2B**). With these selected B cells, we used droplet technology for single-cell recovery and sequencing of the naturally VH : VL paired gene rearrangements that also accurately retained the sequences of the respective CH1 and CL domains (**Figure 2C**).

### Sequence Analysis of Antibody Genes From SpA-Selected B Cells

**Figure 3:**
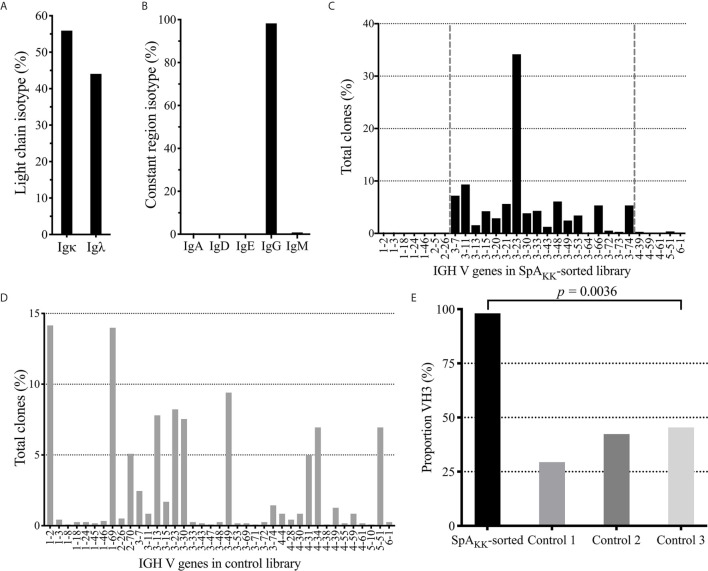
Repertoire analysis of SpA_KK_-sorted memory B cells and unsorted peripheral B cells from healthy controls. **(A)** Isotype usage as a % of total in SpA_KK_-sorted repertoire. **(B)** Light chain usage as a % of total in SpA_KK_-sorted repertoire. **(C)** Gene usage as % of total clones identified in sequenced SpA_KK_-sorted repertoire. Clones mapped to the VH3 gene family are highlighted between the two vertical dashed lines. **(D)** Gene usage as % of total clones identified in the total memory B cell repertoire of a healthy control donor (Control 1). **(E)** Proportion of VH3 encoded BCR in repertoire in the SpA_KK_-sorted peripheral memory B cells compared to three individual healthy controls.

From this single-cell library, we obtained data from an estimated 1,885 individual barcoded cells. Of these individual cells, 1,342 (71.2%) had assignable (i.e., in-frame) VH region contigs, and 1,036 (55.1%) had assignable Vκ region contigs while 831 (44.1%) had assignable Vλ region contigs. Of the 1,342-heavy chain contigs, 752 (39.9%) have VH:Vκ paired sequences, and 578 (30.7%) have VH:Vλ paired sequences resulting in a total of 1,330 individual complete paired heavy and light chain sequences. Notably, from this IgG+ sorted memory B-cell population of productive VH : VL paired sequences, 1,318 (98.28%) included IgG CH1 domain sequences (**Figure 3B**), which attested to the rigor of the selection strategy.

Herein, independent clones were defined as cells expressing unique VH-DH-JH : VL-JL junctional sites that identify a presumed ancestral B cell. Notably, analysis of the antibody gene transcripts from these class-switched selected single CD27+ memory B cells demonstrated that most of these recovered B cells were from unique clones that contained a single cellular representative. However, 43 of these clonal sets included multiple cells with the same VH : VL gene sequences, with the largest clone containing four cells.

Analysis of the VL genes expressed by these SpA_KK_-selected memory B cells demonstrated a modest kappa predominance with a kappa:lambda light chain ratio of 1.27, which approximates the adult physiologic range of kappa light chain predominance (**Figure 3A**) (47). Furthermore, these selected memory B cells expressed rearrangements using diverse germline Vκ gene segments, with the greatest proportion using IGKV1 genes (44.69%) that also presents the largest gene family of inherited Vκ genes. This was followed by IGKV3 (37.74%), IGKV2 (10.57%) families, with 6.56% from the single member gene family, IGKV4, and 0.48% most homologous with the IGKV6 family. Taken together, this usage pattern in memory B cells that bound SpA_KK_, which is devoid of the Fc binding site, is roughly the distribution previously seen in the repertoire of healthy adults (**Supplementary Figure 1A**) (48, 49). These findings document that there was unlikely to have been selection bias in the specific VL usage in the SpA_KK_-binding memory B cells.

### Diverse Clones Bind SpA With Dominant but Not Complete Restricted Usage of VH3 Genes

In order to determine if there was a bias of the superantigen Fab-binding domain we next performed the VH region analysis of SpA_KK_-selected memory cells by identifying the representation of recovered assignable VH genes. Strikingly, we found an overwhelming preference for clones with antibody gene rearrangements assigned to the VH3 family (**Figures 3C, E**), representing 98.08% of assignable full-length heavy chains. By comparison, in large VH-gamma libraries from healthy donors generated using an unbiased RACE based approach (44), VH3 genes were used in 29-45% VH rearrangements (**Figures 3D, E**). This is consistent with earlier reports that the VH3 family, the largest family of heavy chain variable gene segments, generally comprises about 40% of the VH repertoire of peripheral B cells of healthy adults (50, 51). Additionally, the repertoire of mature peripheral B cells generally utilize roughly the same VH gene segment representation as is present in the germline locus (52). Taken together, our single cell sequencing studies documented a significant bias toward VH3 gene usage in SpA-binding class-switched memory B cells (*p* < 0.0001) (**Figure 3E**). The recovery of mainly VH3 family BCRs would suggest a dominance of binding by the superantigen Fab-binding site in the memory B cell selection.

**Figure 4:**
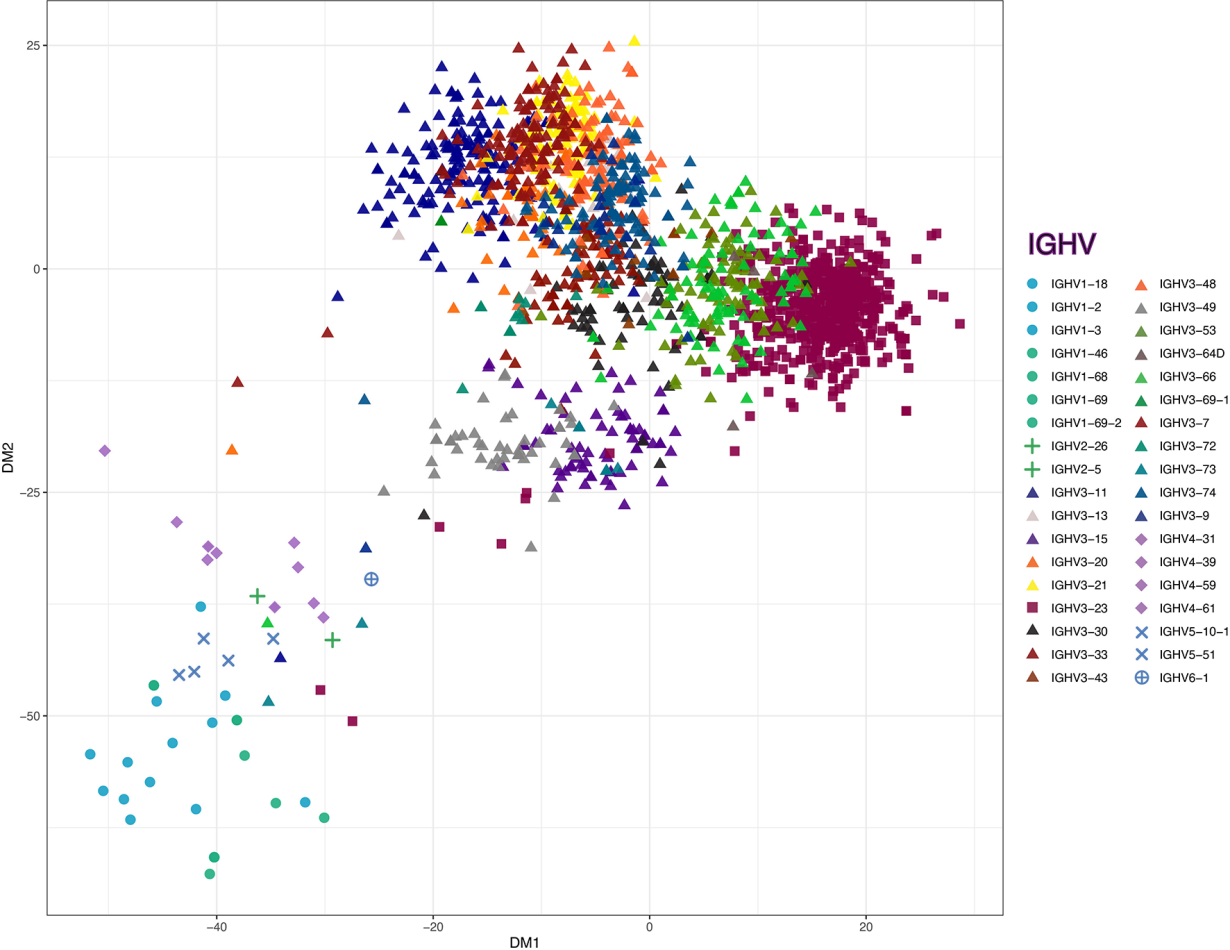
Classical multidimensional scaling on sequence dissimilarity matrix of the VDJ regions from all of the B cell transcripts. Matrix is derived from pairwise longest common substring distance of the all VH sequences from sorted B cells, with dissimilarity between these antibody gene rearrangements depicted in two-dimension. The non-VH3 (circles - VH1, pluses - VH2, diamonds - VH4, ‘X’s - VH5 and sun crosses - VH6), VH3-23 (squares) and VH3-other than from the VH3-23 germline gene (VH3-non23) (triangles) are depicted on the plot. Individual genes are marked with unique colors.

Compared to the representation within the control γ-rearrangement blood B cell cDNA libraries, (**Figure 3D**), sequence analysis revealed that the SpA_KK_-reactive memory B cells also had a strong selection for diverse rearrangements of the VH3-23 gene member of the VH3 family, representing 34.17% of all sorted cells (**Figure 3C**). Interestingly, of the 461 reads assignable to VH3-23 gene rearrangements, these represented 445 unique clones, based on junctional sequences at the V-D-J site representing the combinatorial HCDR3 subdomain (data not shown). To further visualize the VH sequence diversity in these sorted B cells, based on pairwise comparisons of these sequences, we generated a sequence dissimilarity matrix of the VDJ regions of all of these B cell transcripts, which we then depicted as a two-dimensional plot (**Figure 4**). As expected, VH3 transcripts clustered separately from transcripts from other families. Furthermore, the transcripts assigned as VH3-23 gene rearrangements were spatially clustered away from those using other genes within the VH3 family (**Figure 4**). Cumulatively, this spatial distribution of sequence differences, together with the preferential usage of VH3 family members, and especially the VH3-23 gene, define the immunogenetic and structural biases inherent to the nature of these BCR binding interactions with the superantigen Fab-binding domain of SpA.

### Involvement of Putative VH Region Contact Sites for Fab

In a co-crystal of domain D of SpA with a VH3 IgM Fab, this non-immune binding interaction has been shown to be centered on 12 individual contact residues in the VH region (34). As a surrogate measure of the potential structural diversity permissive at this interface, we evaluated the proportion of amino acids at each of these positions with the range of germline VH-encoded residues previously identified at this position for this Fab-SpA contact site (**Supplementary Figure 2**) (34). In brief, we discovered that for 9/12 potential contact residues, representation of non-conservative residues were less than 1.5%. Yet, for the three other potential contact residues, H57, H64 and H68, we found much higher rates, ranging from ~7-15%, which may signify a less prominent, and more permissive, role of these residues in the Fab-mediated SpA binding interaction. As anticipated, at these same positions in the non-VH3 transcripts, there were significantly lower levels of the residues identified at the VH3-SpA interface, compared to the VH3 transcripts, as these residues are not generally conserved at these positions in VH germline genes from different families (34) (**Supplementary Figure 2**). The sequence homology between the BCR transcripts of these selected memory B cells with the reported Fab contact residues strengthens the perceived dominance of the interaction of these membrane-bound BCR with the Fab-binding site on SpA.

### Recognition of Antigenic Sites Other Than the Fab-Binding Site by Memory B Cells

**Figure 5:**
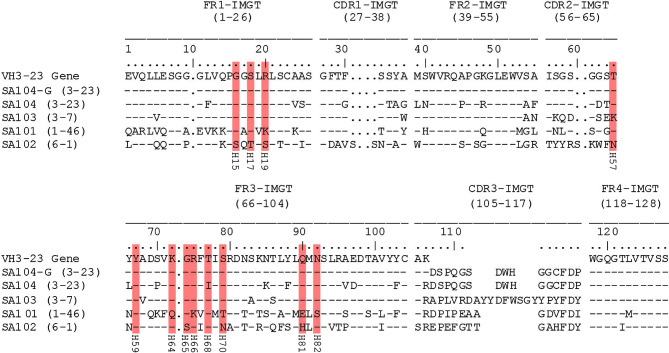
Alignment of heavy chain variable region sequences of mAbs. The deduced amino acid sequences of recombinant monoclonal IgG antibodies are compared to the VH3-23 germline gene sequence using IMGT-number convention. SpA contact residues with the VH region of the antibodies are highlighted with red boxes with the original numbering used in defining the VH3 Fab-SpA binding interaction below each residue. The natural variation in the mAb SA103, T57K is permissive of the binding interaction (34).

Despite the overwhelming number of VH3 family transcripts recovered, we wanted to directly evaluate the binding reactivity and specificity of the SpA_KK_-binding memory B cells, especially those transcripts that were non-VH3. We selected individual representative clones from the VH3 and non-VH3 transcripts, identified by unique paired VH : VL gene sets in the distinct clusters in the sequence dissimilarity matrix (**Figure 4**). We then generated recombinant human IgG1 antibodies, with the IMGT-identified closest germline heavy chain and light chain genes shown in **Supplementary Table 2**. These include antibodies that utilize the VH3-23 gene (mAb SA104), and a distinct VH3 gene (mAb SA103) as well as genes from other families (mAb SA101 and mAb SA102). In part, these mAbs were also chosen as they display differing levels of somatically generated replacement mutations, both in their overall sequences, and at the postulated VH contact residues responsible for non-immune Fab-mediated SpA binding interaction (**Figure 5**) (34, 53, 54). In addition, the VH3-23 gene expressing antibody, SA104, was chosen as it is a highly mutated antibody, which includes a non-conservative replacement mutation in a Fab-SpA contact residue. To further test the potential roles of these sites in this binding interaction, we also generated an antibody (termed mAb SA104-G) that reverted all somatic mutations to germline VH3-23 residues.

**Figure 6:**
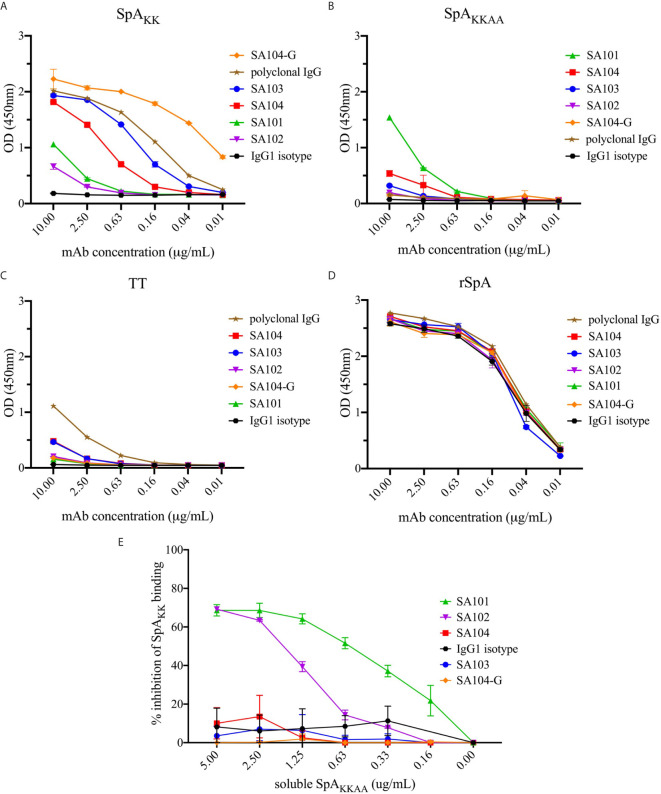
Reactivity of monoclonal antibodies generated from the SpA_KK_-sorted memory B cells. Antibodies generated from identified sequences were tested for binding to SpA variants **(A)** SpA_KK_ protein, **(B)** SpA_KKAA_ protein and **(D)** WT rSpA. **(C)** Tetanus toxoid (TT) was used a control because a subset within polyclonal IgG purified from human serum recognizes this antigen, even though the mAbs do not. While the polyclonal IgG recognized tetanus toxoid due to the high prevalence of anti-TT antibodies in most individuals with up-to-date tetanus shots, the SpA_KK_-selected mAbs minimally bound the unrelated antigen. Each of these mAbs was tested at a range of concentrations from 10 to 0.01 µg/mL. **(E)** Inhibition of individual mAbs binding SpA_KK_ by soluble SpA_KKAA_ is shown as a percentage of total mAb binding reactivity at 1 µg/mL to SpA_KK_.

To determine the reactivity and molecular specificity of these recombinant antibodies for different forms of oligomeric SpA, which measured responses to native wildtype SpA (**Figure 6D**) and SpA_KK_ that is devoid of Fc-binding and used for the initial sort (**Figure 6A**). Notably, because all mAbs were generated as human IgG1 antibodies, the native SpA, which has conserved the Fc-binding site, served as a positive control as all mAbs displayed strong near-identical binding interactions (**Figure 6D**). Within these studies, tetanus toxoid (TT) was used as an irrelevant binding control protein, which was recognized only by a subset of antibodies within the purified polyclonal IgG from human adults, who presumably had received prior TT vaccination (**Figure 6C**). The reconstituted mAbs do not recognize TT.

As anticipated, with the SpA variant that retained the Fab-binding site (i.e., SpA_KK_ variant), the VH3-encoded antibodies, mAb SA103 and mAb SA104 displayed significant dose-dependent binding activity (**Figures 6A, D**). The SA104-G mAb, which included VH region germline reversion mutations, had the strongest reactivity of all tested binding interactions (**Figures 5**, **6A**). Intriguingly, the two non-VH3 mAbs, SA101 mAb and SA102 mAb, which were also selected based on SpA_KK_-tetramer binding activity, displayed significant but relatively weaker SpA_KK_ activity, while the irrelevant isotype control had no detectable binding activity (**Figure 6A**). As a control, we included polyclonal IgG from adult sera, which contains a mixture of antibodies, that displayed intermediate levels of binding activity with the forms of SpA that retained the Fab-binding site (**Figure 6A**).

To identify specificity to other determinants on SpA, we also generated SpA_KKAA_ (**Figure 6B**), a mutant form in which both the Fc-binding and Fab-binding sites are abrogated (**Figures 1B, D**). This SpA variant enabled evaluation of the potential binding reactivity of the experimental mAbs with “conventional” antigenic determinants on SpA that are not mediated by the Fab- or Fc-binding sites. Remarkably, the SA101 mAb bound the SpA_KKAA_ protein (**Figure 6B**). The SA101 mAb conserves only 6/12 of the contact sites identified in the VH3 Fab-SpA interface (**Figure 5**). This evidence of a substantial binding interaction, despite many non-conservative replacement residues in the SpA contact residues, supports the notion that the SA101 mAb recognizes a determinant on the SpA surface distinct from the Fab-binding site. Intriguingly, the other non-VH3 antibody, SA102 mAb, despite having a high load of somatic mutations, displayed binding activity for SpA_KK_ but minimal detectable activity for the SpA_KKAA_ variant (**Figures 6A, B**).

To further evaluate the specificity of these binding interactions to either the Fab-binding site or other determinants, we performed competition immunoassays, in which we preincubated each of these antibodies with titrated concentrations of soluble SpA_KKAA_, and then measured the effect on binding to a fixed concentration of SpA_KK_ coated onto the solid phase. As anticipated, as the VH3 Fab-binding site was absent in SpA_KKAA_, preincubation of soluble SpA_KKAA_ had no effect on the binding interactions of the VH3-encoded antibodies, SA103, SA104, SA104-G. These findings also provide evidence that the SpA mutant, SpA_KKAA_, does not have functional sites that can compete for the binding of these VH3-encoded mAbs (**Figure 6E**). In contrast, soluble SpA_KKAA_ significantly inhibited binding of the SA101 mAb, and less so with the SA102 mAb, to SpA_KK_ coated on the wells, with dose-dependent inhibition. These findings provide strong support that there are determinants on the SpA molecule, separate from the Fab-binding region, that can be targeted by conventional antibody binding interaction (**Figure 6E**).

### Recognition of Diverse Antigenic Sites on SpA by Circulating IgG Antibodies

**Figure 7:**
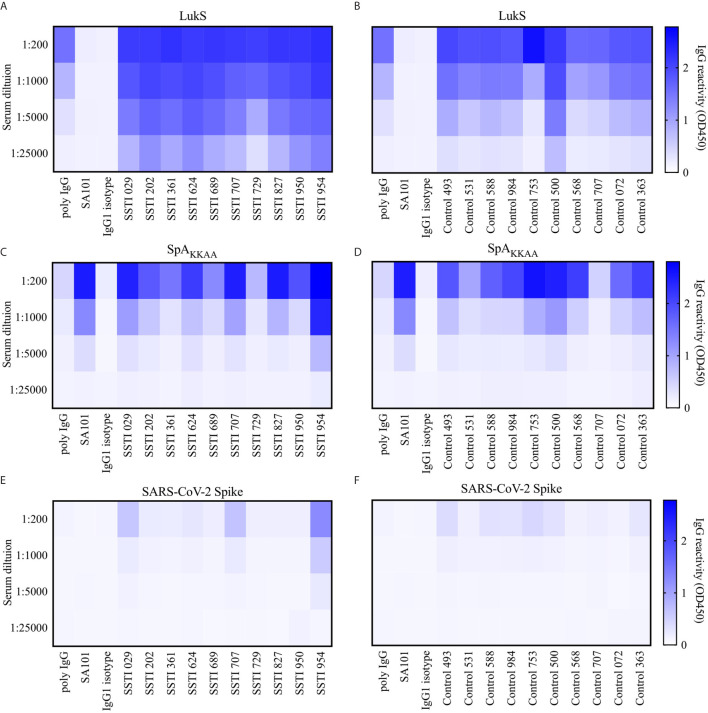
Serum reactivity to Leukocidin S, SpA_KKAA_ and Spike protein in patients with Skin and Soft Tissue Infections (SSTI) and controls. **(A)** Serum binding reactivity measured as OD 450nm to Leukocidin S (LukS) from *Staphylococcus aureus* by individuals with skin and soft tissue infections (SSTI) at 1:200, 1:1000, 1:5000 and 1:25,000 serum dilutions and mAb controls at 10, 2, 0.4 and 0.08 µg/mL. **(B)** Binding reactivity to LukS by control serum and mAb controls. **(C)** Binding reactivity to SpA_KKAA_ by SSTI sera and mAb controls. **(D)** Binding reactivity to SpA_KKAA_ by control serum and mAb controls. **(E)** Binding reactivity to Spike protein from SARS-CoV-2 by SSTI sera and mAb controls. **(F)** Binding reactivity to Spike protein by control serum and mAb controls.

To extend the above-described diverse SpA determinant-binding properties of the mAbs, we next sought to investigate the binding specificities of circulating antibody responses in both *S. aureus*-infected and healthy adults. We therefore characterized the binding interactions with determinants on the SpA protein, apart from the Fab- and Fc-binding regions, with serum IgG from individuals with *Staphylococcus aureus* Skin and Soft Tissue Infections (SSTI) and healthy adults (**Figure 7**). SSTI is a common form of *S. aureus* infection, which involves the skin, muscles, and connective tissue such as ligaments and tendons, that generally requires antibiotics for resolution (55, 56). Moreover, these infections generally result in enhanced B-cell and antibody responses to a range of staphylococcal products, that in most cases peaked at about 6 weeks (12, 15, 16, 45).

In studies of ten representative individuals recovering from SSTI at the peak of their responses, and in ten healthy controls, we assessed antibody levels against Leukocidin S (LukS), a *S. aureus* pore-forming toxin as an infection control. We found a significantly more robust IgG anti-LukS response than was demonstrated in control sera (**Figures 7A, B** and **Supplementary Figure 4A**). Interestingly, we found that most individuals also displayed robust levels of IgG binding to the SpA_KKAA_ mutant (**Figure 7C**) although this was at a significantly lower level than the LukS targeted response (**Supplementary Figure 4D**). Notably, we also found high levels of circulating IgG anti-SpA_KKAA_ antibodies, but these were less than the LukS response, in sera of healthy adults without recent infection (**Figures 7B**, **D** and **Supplementary Figures 4B, D**). Hence, most healthy adult individuals have robust levels of circulating antibodies to SpA_KKAA_.

To determine the relative magnitude of these antibody responses, these same assays were repeated using the recombinant Spike protein from SARS-CoV-2, and these same clinical sera that were obtained years before the recent COVID-19 pandemic. Hence, the donors were anticipated to be immunologically naïve of the Spike protein. As anticipated, only rare subjects displayed any binding signal for the Spike protein (**Figures 7E, F** and **Supplementary Figure 4C–E**). Importantly, for serum IgG from both the SSTI and healthy donors the binding reactivity for SpA_KKAA_ was significantly stronger than the binding reactivity for the SAR-CoV-2 Spike (*p* = 0.0020, **Supplementary Figure 4D–E**). Indeed, the SSTI and control donors displayed the same patterns of binding reactivity with either SpA_KKAA_ or the COVID-19 Spike protein (**Supplementary Figures 4B, C**). In contrast, when compared to responses in healthy adults there were significantly higher levels of binding reactivity against LukS in the infected group at 6 weeks after infection onset (**Supplementary Figure 4A**). These findings suggest that the surface recognized on SpA_KKAA_ by non-VH3 antibodies, such as SA101, may not behave entirely akin to a conventional antigen, such as LukS, for which recent immune exposure to *S. aureus* boosts the serum IgG anti-toxin antibody response. Taken together, our serological studies confirm the existence of an adaptive immune response to other conventional antigenic determinants on SpA, which can be detected, albeit at low levels, in the circulation of both *S. aureus*-infected and healthy adults.

## Discussion

SpA is a virulence factor with a highly stable triple α-helical bundle structure, upon which two well-defined sites, for Fc- and Fab-binding, have presumably arisen during the co-evolution of this host-pathogen relationship. During clinical infection, interactions with these sites modulates host immune defenses (23, 57). The Fab-binding site has optimal interactions with a substantial proportion of germline-encoded BCR/Ig (58), which therefore favor binding of peripheral naïve mature B cells, especially from human neonates, and also innate-like B cells (Marginal zone and B-1) as shown in mice (59). Our current studies provided an in-depth examination of the relationships, defined by binding interactions, between human memory B cells with SpA.

Toward this goal, we developed a method to sort for relevant antigen-specific memory B cells using SpA-antigen-tetramers, which was used to investigate the BCR-mediated binding interactions of class-switched peripheral memory B cells. To ensure that memory B cells were selected solely by BCR-mediated selection, we used an engineered version of SpA that abrogated Fc-binding ability, termed SpA_KK_ (27). Despite the potential for non-immune interactions, we reasoned that mammalian immunity should still be capable of immune recognition beyond the toxin-associated Fc- and Fab-binding sites. These binding interactions of B cell clones are postulated to have arisen from recurrent immune exposure to *S. aureus* antigens, during the many infections and exposures suffered through the years. In specific, we document BCR-mediated specificity to extra-toxin determinants within the repertoire of both peripheral memory B cells, and circulating antibodies that are the presumed *in vivo* products of plasma cells.

Previous work has postulated that SpA contains non-VH3 restricted conventional epitopes (60), which are accessible on SpA_KKAA_ mutant sequence variations that are designed to remove the Fab-contact residues elucidated with an Fab/SpA co-crystal (34). However, this work has been performed in experimental mice that are naïve to *S. aureus* antigens, whereas humans are generally not. Thus, there has been a major interest in elucidating the role of SpA and its interactions with B cells in large part to understand the immunologic consequences of *S. aureus* infection in humans (21). While only a small proportion of the sorted memory B cells represented non-VH3 gene encoded BCR, representative VH : VL transcripts were constructed as full-length antibodies, SA101 and SA102 (**Table S2**), which admittedly displayed lessor SpA_KK_-binding activity when compared to the VH3 mAbs, SA103 and SA104 (**Figure 6A**). However, when tested for interactions with a mutant form of SpA with both the Fab and Fc sites removed (SpA_KKAA_), we found that one of the non-VH3 mAbs, SA101, bound this mutant SpA form, *via* a determinant on the molecule functionally distinct from the Fab- or Fc-binding sites (**Figure 6B**). This binding was confirmed by demonstration that soluble SpA_KKAA_ can inhibit 80% of SA101 mAb binding activity for SpA_KK_ that is devoid of only the Fc binding site (**Figure 6E**). While relatively less frequent than VH3-encoded B cells, we were able to identify the presence of memory B cells in circulation that can recognize other epitope determinants on the SpA molecule.

The recognition of these “alternative” SpA determinants was clearly demonstrated with a variant form of SpA in which the side chains of only four amino acids (of the 56-61 amino acids in native domain D) were substituted (34, 46), which based on modelling (**Figure 1B**), affected only 7.65% of the exposed solvent-accessible molecular surface. Moreover, these changes were predicted to not otherwise affect solubility of folding of these pentameric constructs (**Figure 1**) (27). In contrast, traditionally employed methods for epitope-identification, such as alanine-scanning, may disrupt the stability of the triple-alpha helical bundle structure of each SpA domain, and therefore may not be suitable for studies of discontinuous sites in a SpA domain. Crystallographic analyses that defined the structures of these domains, in complex with Fab and Fc molecules (34, 46), were also beyond the scope of this report, but could be applied in future studies of the binding interactions with these mAbs. It nonetheless would be interesting to see if the binding of the mAb SA101 with SpA_KKAA_ involves residues within the Fab- or Fc-binding sites. Whereas our studies are the first to directly demonstrate that there is a detectable set of alternative-determinant binding by B-cell clones and circulating antibodies in the human immune system (**Figures 6**, **7**), further studies are required, in part to measure relative affinities and kinetics of binding of the mAbs to SpA variants, and to further localize contact sites.

The antibody transcripts of the sorted cells were identified using single-cell sequencing technology and VH-VL analysis detected use of diverse VL genes but was highly biased (i.e. 98.5%) to the usage of genes from the VH3 family, a unique binding characteristic of SpA that was previously described (34). In earlier flow cytometric surveys, the peripheral blood total mature B cells of healthy adults had a very narrow physiologic range (i.e., mean 30+/-2%) of Fab-mediated binding to a fluorochrome-labeled form of SpA (58). The cumulative literature indicates that these interactions result from recognition of a conserved conformational surface on VH regions encoded by structurally related genes of the VHIII clan that are highly represented in almost all mammalian immune systems (34, 61–64). Our study therefore demonstrated highly significant (*p* < 0.00001) recovery of a VH3-enriched set of B cells and our findings therefore confirmed the selection of SpA-binding switched post-germinal center B cells that was solely based on the VH region and was apparently not affected by light chain usage. Furthermore, a high proportion (34.17%) of these selected VH regions were identified as rearrangements of the VH3-23 gene (**Figure 3C** and **Supplementary Figure 3**). These finding confirmed results from long ago, when we used recombinant SpA to select for Fab binders using a Fab-phage-display library system that we generated with VH-gamma and Kappa/Lambda light chain rearrangements from a healthy donor. Through sequential competitive rounds of selection, we isolated SpA binders with a range of affinities. We thereby demonstrated the expected specificity for VH3-encoded Fab, as well as documented that the strongest binding, was with clones encoded by the VH3-23 gene (previously termed VH26) (65, 66). Intriguingly, at a nucleic acid sequence level and at an amino acid sequence level, the VH3-23 gene is the closest to the overall consensus sequence of all members of this family (**Supplementary Figure 3**). This binding interaction is conserved across species presumably due to co-evolution of this bacteria with mammalian hosts across the eons (67).

In our current studies, we confirmed the structural basis for *in vivo* binding of SpA by generating full-length antibodies from the selected memory B cell BCR. Other than a natural T57K germline VH3 gene-associated variation, the mAb SA103 is devoid of non-conservative replacement amino acids at the SpA Fab contact residues (**Figure 5**). As expected, this mAb had the strongest binding activity of all mAbs selected from the transcripts (**Figure 6A**). This was followed by the SA104 mAb, which was highly mutated throughout the variable region chain, including a mutation, T68I, in a contact residue (**Figure 5**), which as a consequence did not bind as strongly as SA103 (**Figure 6A**).

To further test this hypothesis, we created a mutated SA104 mAb, in which reversion mutations to the VH3-23 germline gene were introduced into the heavy chain variable region. The resulting SA104-G mAb displayed a robust increase in binding reactivity for SpA_KK_ when compared to the originally somatically mutated form of the SA104 mAb (**Figure 6A**). These results therefore reaffirm the preeminence of VH3 germline-encoded antibodies in Fab-mediated SpA binding, and the great enrichment for VH3-23 encoded antibodies in the SpA-selected library can only be explained if the VH3-23 gene encodes the optimal VH surface for these non-immune SpA binding interactions. Our sampling of thousands of selected memory B cells suggested that even the immense potential power for clonal selection and somatic remolding of BCR could not out-compete binding to the relatively limited Fab-binding site on SpA, which is estimated to represent less than 8% of the surface of the molecule. The power of these interactions is the source of the superantigen influence of SpA on *in vivo* B cell clonal selection of differentiated plasma cells (68). Implicit to our studies is the model that from birth there is continuous generation of new B cells in the bone marrow expressing VH3-germline genes, which represent an astonishingly high frequency of potentially SpA-reactive B cells. During *S. aureus* infection, these B cells would be predicted to dominate clonal competition for SpA interactions in follicular (i.e., germinal center) or extrafollicular sites (18). We wonder whether the bias for these VH3 B cells, which is encoded in the germline of inherited VH gene segments, could be considered the equivalent of a “pre-original super-antigenic sin”.

Our findings have great clinical relevance for vaccine development. Whereas earlier reports have been limited to murine studies (18, 27, 60), the current work is the first to document the antigenicity of alternative determinants on SpA for the human immune system. We have recently reported the molecular characterization of immunogenic sites in staphylococcal Leukocidins, which induce *in vivo* responses that can contribute to functional inactivation of these important toxins (69, 70). Akin to our advocacy of these minimal epitopes on Leukocidins for inclusion in multi-module vaccines, our current studies herald a practical approach towards further targeted augmentation of immunity *via* vaccination by inclusion of alternative immunogenic SpA sites. Such an approach, which would expand B-cell clones recognizing these determinants, would be predicted to pre-condition the host for further preferentially expansion as a consequence of re-exposure to SpA produced *in vivo* during clinical infection. Such a redirected immune response should provide host protection by enhanced clearance of this highly produced toxin from the earliest stages of infection. Such a vaccine would also be predicted to strongly enhance host immune responses to many other staphylococcal virulence factors, and thereby augment defense from potentially many types of *S. aureus* clinical syndromes.

## Data Availability Statement

The datasets presented in this study can be found in online repositories. The names of the repository/repositories and accession number(s) can be found below: https://www.ncbi.nlm.nih.gov/sra/PRJNA694313, PRJNA694313.

## Ethics Statement

The studies involving human participants were reviewed and approved by Institutional Review Board (IRB). The patients/participants provided their written informed consent to participate in this study.

## Author Contributions

GS and ER conceived and designed the experiments. ER, ZL, DH, HE, and SP performed the experiments. ER wrote the manuscript. ZL, HE, DH, BS, SP, PL, DF, and GS performed data analysis and revised the manuscript. All authors contributed to the article and approved the submitted version.

## Funding

This work was supported through the NIH/NIAID contract HHS N272201400019C, “B Cell Epitope Discovery and Mechanisms of Antibody Protection,” and T32 GM066704 (ER) and the Colton Foundation (GS).

## Conflict of Interest

The authors declare that the research was conducted in the absence of any commercial or financial relationships that could be construed as a potential conflict of interest.
