# Supplementary material for: Diversity of Functionally Distinct Clonal Sets of Human Conventional Memory B Cells That Bind Staphylococcal Protein A
Source: Front Immunol. 2021 Apr 28;12:662782. doi: 10.3389/fimmu.2021.662782 (PMC8113617; doi:10.3389/fimmu.2021.662782)
Supplement: Supplementary file 1 [file DataSheet_1.docx]

**Supplemental Figures and Tables:**


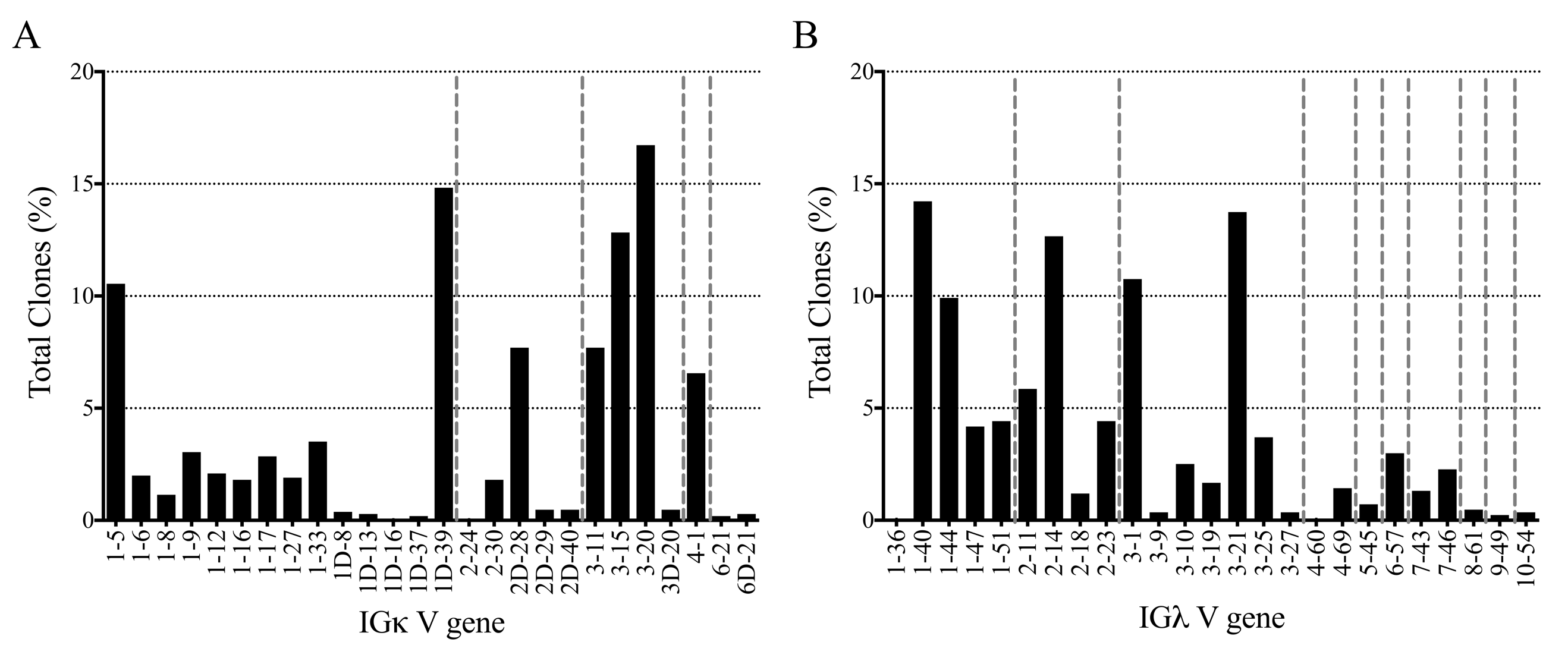


**Supplementary Figure 1:** Light chain gene usage. **(A)** Vκ gene assignments based on homology to the closest known human kappa germline gene segment. Representation in the repertoire is depicted as a percentage of the total clones identified. **(B)** Vλ gene assignments based on homology to closest known human lambda germline genes. Genes from different gene families are segregated with a vertical grey dotted line.


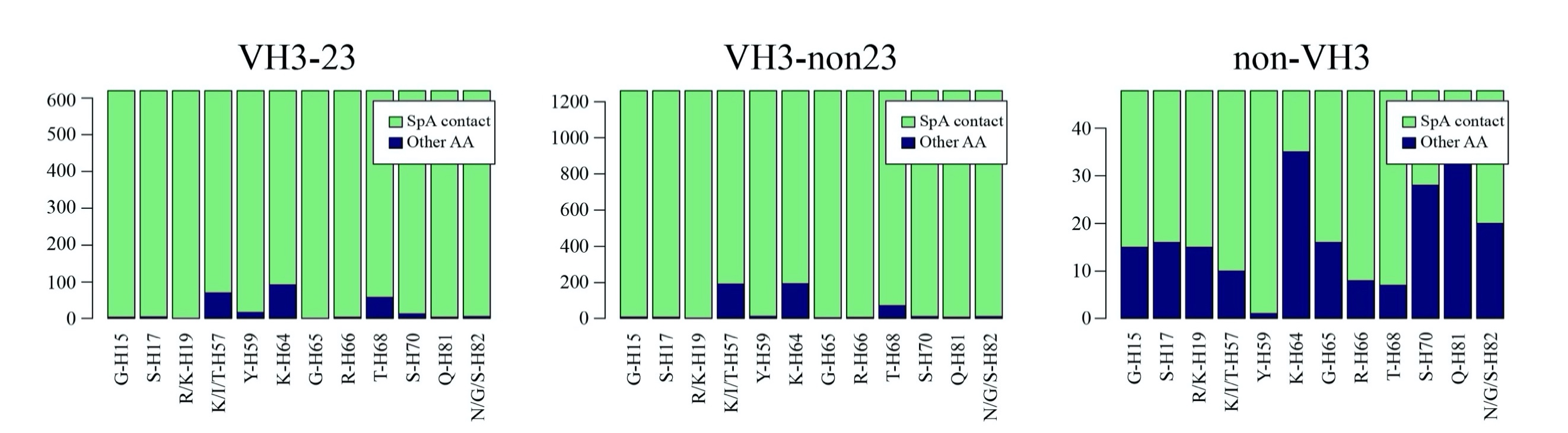


**Supplementary Figure 2:** Frequency of permissive amino acids at key residue sites that allow for SpA-Fab contact in antibody transcripts from the SpA_KK_-sorted memory B cells. The contact residues between the Fab-binding domain of SpA and the Fab have been previously identified (Graille et al.). Prevalence of amino acids that are key contact residues between the Fab VH region and the Fab binding site of SpA in different groups of antibody transcripts are identified in green at each position, presumed non-binding amino acids at each position are shown in blue. The VH3-23 and VH3-non23 antibody transcripts have similarly high levels of interacting amino acids at the contact residues whereas the non-VH3 antibody transcripts have higher rates of different amino acids at most residues.


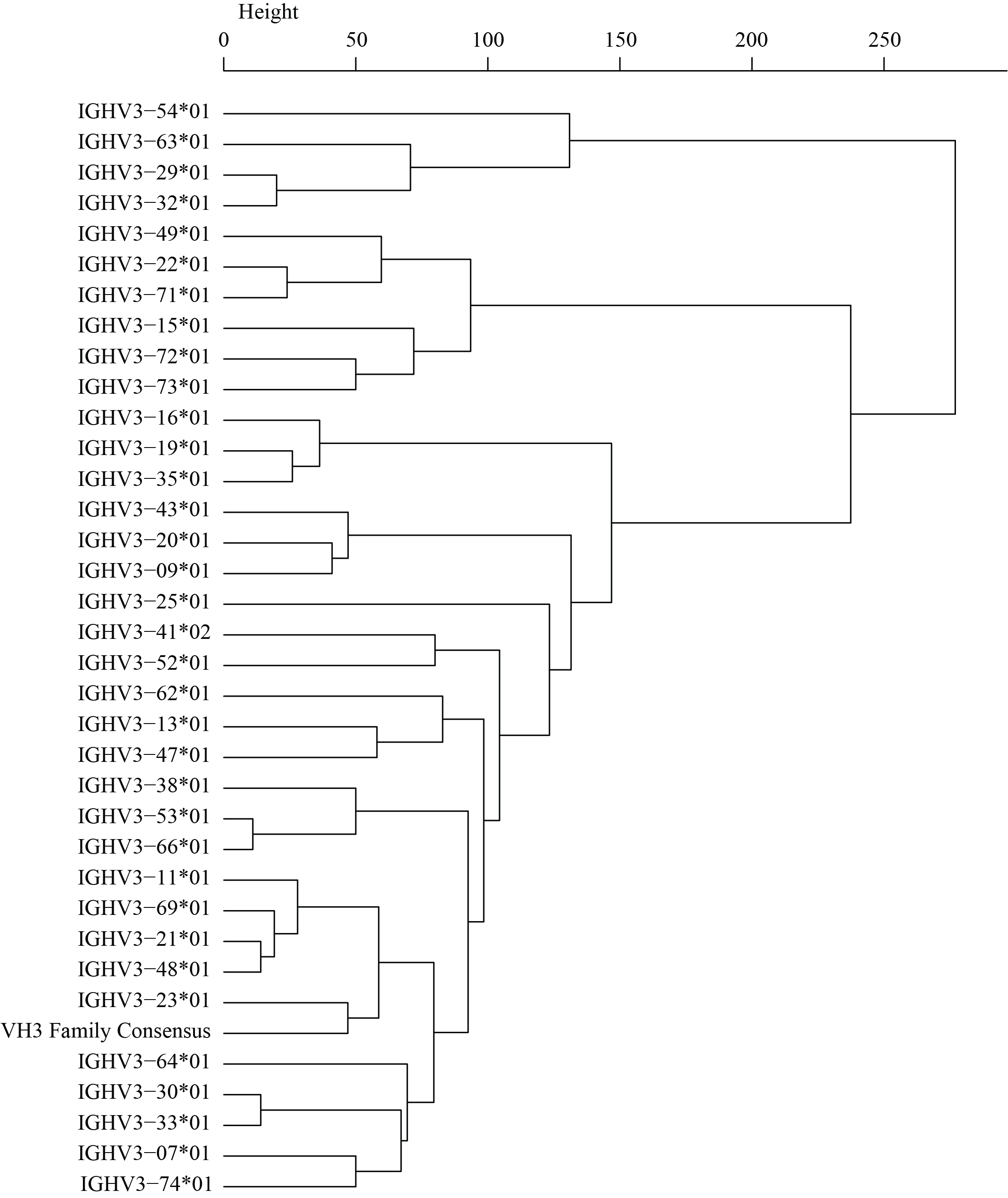


**Supplementary Figure 3:** Dendrogram of VH3 family member sequence identity**.** Germline sequences for each VH3 family member are clustered based on sequence identity on the germline configuration. The VH3-23 gene is most closely clustered with the overall VH3 family consensus.


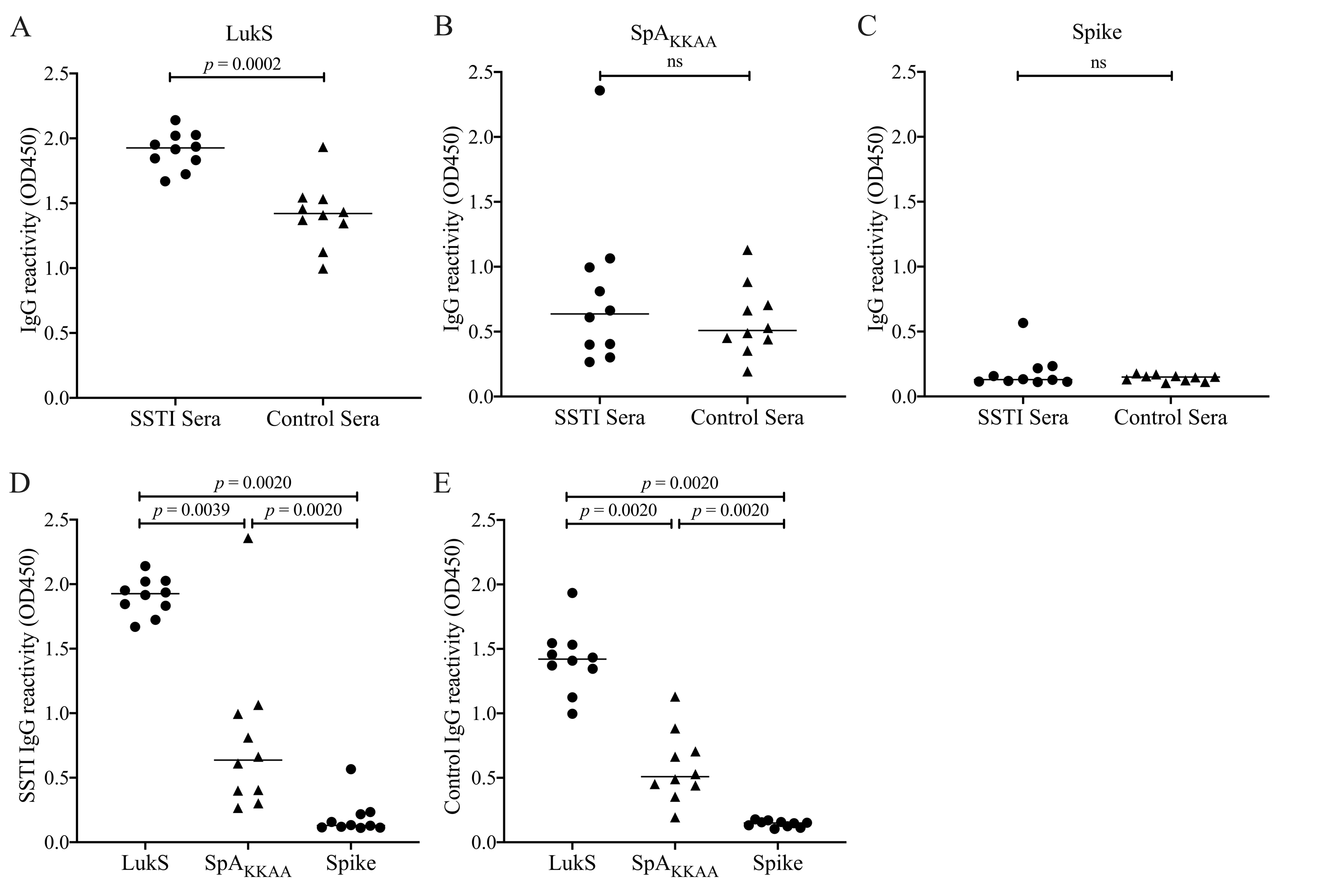


**Supplementary Figure 4**: Differences in IgG anti-LukS, SpA_KKAA_ and Spike reactivity in SSTI or control sera. IgG reactivity in serum was measured with binding against LukS, SpA_KKAA_ and Spike at a serum dilution of 1:1000 for either SSTI or control sera. **(A)** IgG reactivity between SSTI sera and control sera binding LukS. SSTI sera has a significantly higher IgG reactivity against LukS than control sera as measured by a Mann-Whitney t test. **(B)** IgG reactivity between SSTI sera and control sera binding SpA_KKAA_. There is no significant difference in IgG reactivity between SSTI or control sera binding SpA_KKAA_ as measured by a Mann-Whitney t test. **(C)** IgG reactivity between SSTI sera and control sera binding Spike protein. There is no significant difference in IgG reactivity between SSTI or control sera binding Spike as measured by a Mann-Whitney t test. **(D)** Differences in SSTI serum binding reactivity to either LukS, SpA_KKAA_ and Spike protein. Wilcoxon t tests were used to determine significance between the paired measurements. **(E)** Differences in control serum binding reactivity to either LukS, SpA_KKAA_ and Spike protein. Wilcoxon t tests were used to determine significance between the paired measurements.

**Supplementary Table 1: Flow cytometry panel of antibodies to identify SpA-binding CD27+ IgG+ memory B cells**

| Marker: | Fluorophore: | Vendor: | Catalog #: |
| --- | --- | --- | --- |
| SAV-SpA_KK_ | PE | ThermoFisher | S21388 |
| SAV-HSA | PE*AF647 | ThermoFisher | S20992 |
| CD19 | APC-Cy7 | BD Biosciences | 557791 |
| CD27 | FITC | BD Biosciences | 555440 |
| CD3 | PerCP-Cy5.5 | Biolegend | 300328 |
| CD14 | PerCP-Cy5.5 | BD Biosciences | 561116 |
| IgG | V450 | BD Biosciences | 561299 |
| IgD | BV605 | Biolegend | 348232 |
| CD38 | PE-Cy7 | Biolegend | 303516 |
| Live/Dead | DAPI | Biolegend | 422801 |
| Mouse IgG1 isotype | Blocking | ThermoFisher | MA1-10407 |

**Supplementary Table 2: Antibodies selected for further binding characterization.** Gene usage is shown for each V(D)J region of the heavy and light chain.

| **mAb name:** | **HV gene** | **HD gene** | **HJ gene** | **LV gene** | **LJ gene** |
| --- | --- | --- | --- | --- | --- |
| SA101 | IGHV1-46*01 F  (91.67%) | IGHD6-19*01 F (RF2) | IGHJ3*02 F (98.00%) | IGKV1-39*01 F (91.76%) | IGKJ3*01 F (94.74%) |
| SA102 | IGHV6-1*01 F (93.94%) | IGHD1-1*01 F (RF1) | IGHJ4*02 F (91.67%) | IGKV4-1*01 F (96.97%) | IGKJ3*01 F (92.11%) |
| SA103 | IGHV3-7*01 F (100%) | IGHD3-3*01 F (RF2) | IGHJ4*02 F (97.92%) | IGKV1-38*01 F (98.92%) | IGKJ1*01 F (92.11%) |
| SA104 | IGHV3-23*01 F (84.38%) | IGHD6-19*01 F (RF1) | IGHJ5*02 F (90.20%) | IGKV3-20*01 F (93.97%) | IGKJ2*02 F (88.89%) |
| SA104-G | IGHV3-23*01 F (100%) | IGHD6-19*01 F (RF1) | IGHJ5*02 F (90.20%) | IGKV3-20*01 F (93.97%) | IGKJ2*02 F (88.89%) |
